# Application of online case-based learning in the teaching of clinical anesthesia for residents during the COVID-19 epidemic

**DOI:** 10.1186/s12909-021-03047-2

**Published:** 2021-12-09

**Authors:** Yi Duan, Zuozhi Li, Xiaoyu Wang, Zhifeng Gao, Huan Zhang

**Affiliations:** 1 Department of Anesthesiology, Beijing Tsinghua Changgung Hospital, School of Clinical Medicine, Tsinghua University, No. 168 Litang Road, Beijing, 102218 China; 2 Department of Special Care Center, National Clinical Research Center for Cardiovascular Diseases, Fuwai Hospital, National Center for Cardiovascular Diseases, Chinese Academy of Medical Sciences and Peking Union Medical College, Beijing, 100037 China

**Keywords:** Case-based learning, WeChat, Anesthesia education

## Abstract

**Objective:**

COVID-19 prevention and control demand a reduction in crowd gathering, which has a significant impact on traditional teaching and offline case-based learning (CBL). In order to mitigate the impact of the COVID-19 outbreak on clinical teaching, we aimed to compare the effects of an online CBL with traditional teaching model on learning outcomes of anesthesia residents.

**Methods:**

Residents rotated in the Department of Anesthesiology in Beijing Tsinghua Changgung Hospital from January 2020 to February 2021 were included in Group W (*n* = 19), which implemented the W-CBL teaching model. The performance of residents was evaluated with theory test and 2 survey questionnaires (A and B) were conducted after 1 month of rotating. All 20 residents rotating in the Department of Anesthesiology at our hospital from January 2018 to December 2019 were included in Group C, which implemented the traditional teaching model. Their examination results were acquired through the teaching files and survey questionnaire (A) were administered through WeChat.

**Results:**

During the 1-month rotation, a total of 10 cases were discussed in Group W. The average score for theory test was higher in Group W than that in Group C (84.57 ± 4.87 vs. 79.35 ± 3.70, *P* = 0.001). The satisfaction rate was also in favor of Group W regarding to clinical thinking, communication skills, learning interest and self-learning ability (*P* < 0.05).

**Conclusions:**

Online CBL based on WeChat platform is an effective and acceptable teaching strategy in comparison to lecture-based learning (LBL) among residents embarking on clinical anesthesia courses.

## Introduction

Since February 2020, in response to the requirement of “reducing non-essential gatherings” for the prevention and control of the COVID19 outbreak, offline lectures and skill training sessions for residents have been cut down on a large scale in mainland China [1]. Some residents are unable to receive training at the hospital due to exposure history of endemic areas. At the same time, the hospital has implemented a fully booked outpatient clinic system, resulting in a significant reduction in inpatient admissions. These factors have a great impact on the traditional clinical teaching model [2].

Traditional anesthesia teaching is based on offline lecture-based learning (LBL), which has limitations in terms of learning drive, independent learning ability and critical thinking exercise [3–5]. Case-Based Learning (CBL) takes clinical cases as a background and encourages residents to think actively as medical experts. Studies have shown that CBL promotes knowledge assimilation, strengthens critical thinking training, and helps enhance residents’ professional competency [6, 7]. However, the preparation of traditional CBL is relatively time-consuming, and the number and variety of teaching cases are relatively homogeneous, which is not beneficial to maintaining residents’ interest in learning [8, 9]. The high aggregation requirements of traditional CBL for trainees also make it difficult to implement and promote in clinical teaching, especially in the regular epidemic prevention and control phase. To tackle these problems, we tried to bring the traditional CBL online. We believe that with the help of the internet and mobile devices, it is possible to break through the physical and temporal constraints of clinical teaching.

Social platforms such as Facebook and Twitter have been introduced into medical education with great success [5, 10]. Being an important achievement in the era of the Internet, instant messaging software has developed into an integrated information platform. Although WeChat was initially designed instant messaging, it now has integrated more sophisticated functions such as communication, messaging, search and office collaboration. It has many advantages of social platform. Given that WeChat already has more than 1.2 billion users, this accessibility gives it a greater advantage in real-time communication and attendance supervision [11, 12]. In recent years, it has been thought that WeChat may help improve the efficiency of medical education [13–16]. WeChat, as the most common online communication platform in China, may eliminate physical limitations in medical education and is a suitable medium for online teaching, the effectiveness of which has been proven in flipped classrooms. However, studies focusing on the use of WeChat combined with CBL (W-CBL) in anesthesia teaching remains scarce. This study aims to investigate the feasibility and acceptability of an online CBL based on a WeChat platform for resident teaching of clinical anesthesia.

## Materials and methods

### Participants

Residents rotating in the Department of Anesthesiology at a tertiary teaching hospital from January 2020 to February 2021 were enrolled in Group W, which implemented the W-CBL teaching model. All 20 residents rotating in the Department of Anesthesiology at our hospital from January 2018 to December 2019 were included in Group C, which implemented the traditional teaching model.

### Teaching strategy

All residents in Group W had their own cell phones and were required to install WeChat. Residents and mentors were trained to use WeChat to participate in CBL. We created a WeChat group as an online CBL communication platform. 4–5 residents who entered the anesthesiology rotation in the same month and 2 mentors joined the same WeChat group.

The steps of classical CBL include topic selection, sequence questions, discussion, evidence interpretation and clinical decision making. In order to give full play to the dual advantages of instant messaging and CBL, the W-CBL teaching method adopted in this study is appropriately modified on the basis of classical CBL. Residents selected CBL cases on their own under the guidance of their mentors and uploaded clinical information (including pre-anesthesia evaluation, consultation records, anesthesia records and prognosis) in the WeChat group. The group members discussed and voted on whether the case was selected as a CBL case, and the mentor was responsible for reviewing the case.

The group members discussed the CBL cases and summarized the problems within a set number of days. The categorized questions were assigned by the mentor to the residents in the group, who searched for information, summarized the answers on their own, and posted their insights in the group via text, image, voice, video, or other forms.

Mentors participated in CBL discussions and provide guidance to residents when necessary. The role of the mentor was to guide the group in deeper and broader thinking, as well as to ensure that each group member was involved in the discussion.

### Teaching evaluation and assessment

Teaching evaluations focused on assessing residents’ knowledge and skills related to clinical anesthesia, and whether their critical clinical thinking and teamwork skills have improved. Teaching evaluation was divided into two parts: (1) assessment at the end of the rotation, including theoretical and practical test to assess teaching effectiveness. Group W had a test after 1 month of rotation. The examination results of Group C were obtained from the teaching files for data analysis. (2) Residents’ self-administered questionnaires were used to collect their perceptions. Two questionnaires were conducted in this study. Residents in both groups were surveyed about overall teaching satisfaction (Questionnaire A), and in addition, residents in the W group were surveyed about acceptance of W-CBL (Questionnaire B). The questionnaire was designed and modified according to previous studies and the purpose of this study, with its validity verified in a previous study [17–19].

### Statistics

Data analysis was done using SPSS 26.0. The mean and standard deviation were used for continuous variables, and frequencies and percentages were used for categorical variables. In this study, nonparametric tests were performed for continuous variables such as age and test scores (normal distribution). Chi-square test was used for classification variables such as gender (male/female) and satisfaction (satisfied/dissatisfied). Differences were considered statistically significant when *P* < 0.05.

## Results

A total of 39 residents were included in this study, including 19 residents in group W and 20 residents in group C. No significant differences were observed in their baseline data Between the two groups (Table 1).

**Table 1:** Basic information of residents in both groups

	W (*n* = 19)	C (*n* = 20)	Statistical value	*P* value
Age (yr)	26.47 ± 2.34	27.95 ± 2.25	−2.002	0.053
Gender			0.022	0.882
Male [n (%)]	9 (47.3)	9 (45.0)		
Female [n (%)]	10 (52.7)	11 (55.0)		
Specialty			0.244	0.621
Anesthesiology [n (%)]	8 (42.1)	10 (50.0)		
Other [n (%)]	11 (57.9)	10 (50.0)		

At the end of the study, a total of 10 cases were discussed in Group W and the theoretical test scores of group W were significantly higher than those of group C (84.57 ± 4.87 vs. 79.35 ± 3.70, *P* = 0.001). However, clinical practice skill test scores were comparable between the two groups (*P* > 0.05) (Table 2). A total of 10 case discussions were completed by Group W during the rotation. Questionnaires (Survey B) were distributed to residents in group W at the end of the rotation to assess the acceptance of WCBL. Nineteen questionnaires were distributed and 19 were recovered, with a 100% valid recovery rate (19/19) (Table 3).

**Table 2:** Assessments of residents in both groups

	W (*n* = 19)	C (*n* = 20)	Statistical value	*P* value
theoretical test				
Before the start of rotation	60.26 ± 6.44	60.90 ± 8.79	−0.257	0.799
At the end of rotation	84.57 ± 4.87	79.35 ± 3.70	3.711	0.001
clinical skill test				
Before the start of rotation	61.05 ± 6.76	62.10 ± 8.36	−0.429	0.671
At the end of rotation	77.37 ± 5.08	78.60 ± 4.59	−0.795	0.432

**Table 3:** The results of questionnaire focusing on W-CBL at the end of rotation

Questions	Yes N (%)	No N (%)
1. Did you enjoy discussing the CBL cases?	15 (78.9)	4 (21.1)
2. Do you appreciate online CBL communication via WeChat?	15 (78.9)	4 (21.1)
3. Did you participate in the group discussion?	16 (84.2)	3 (15.8)
4. Could you communicate ideas as effectively as possible in the group discussion?	13 (68.4)	6 (31.6)
5. Were you able to complete the tasks assigned to you on time?	15 (78.9)	4 (21.1)
6. Could you employ your acquired knowledge to solve problems?	13 (68.4)	6 (31.6)
7. Could you contribute additional information to the group discussion?	10 (52.6)	9 (47.4)
8. Could you raise questions freely in the group discussion?	14 (73.7)	5 (26.3)
9. Did you notice the intrinsic connection of the information?	11 (57.8)	8 (42.2)
10. Could you critically evaluate the knowledge you obtained?	13 (68.4)	6 (31.6)
11. Did your mentor behave enthusiastically in W-CBL?	14 (73.7)	5 (26.3)
12. Did your group members and mentors provide timely feedback to enhance your learning?	15 (78.9)	4 (21.1)
13. Do you think WeChat can improve the effectiveness of CBL in the training of anesthesiology?	14 (73.7)	5 (26.3)
14. Do you agree that an online CBL based on the WeChat platform can improve the clinical competency of residents?	16 (84.2)	3 (15.8)

**Fig. 1:**
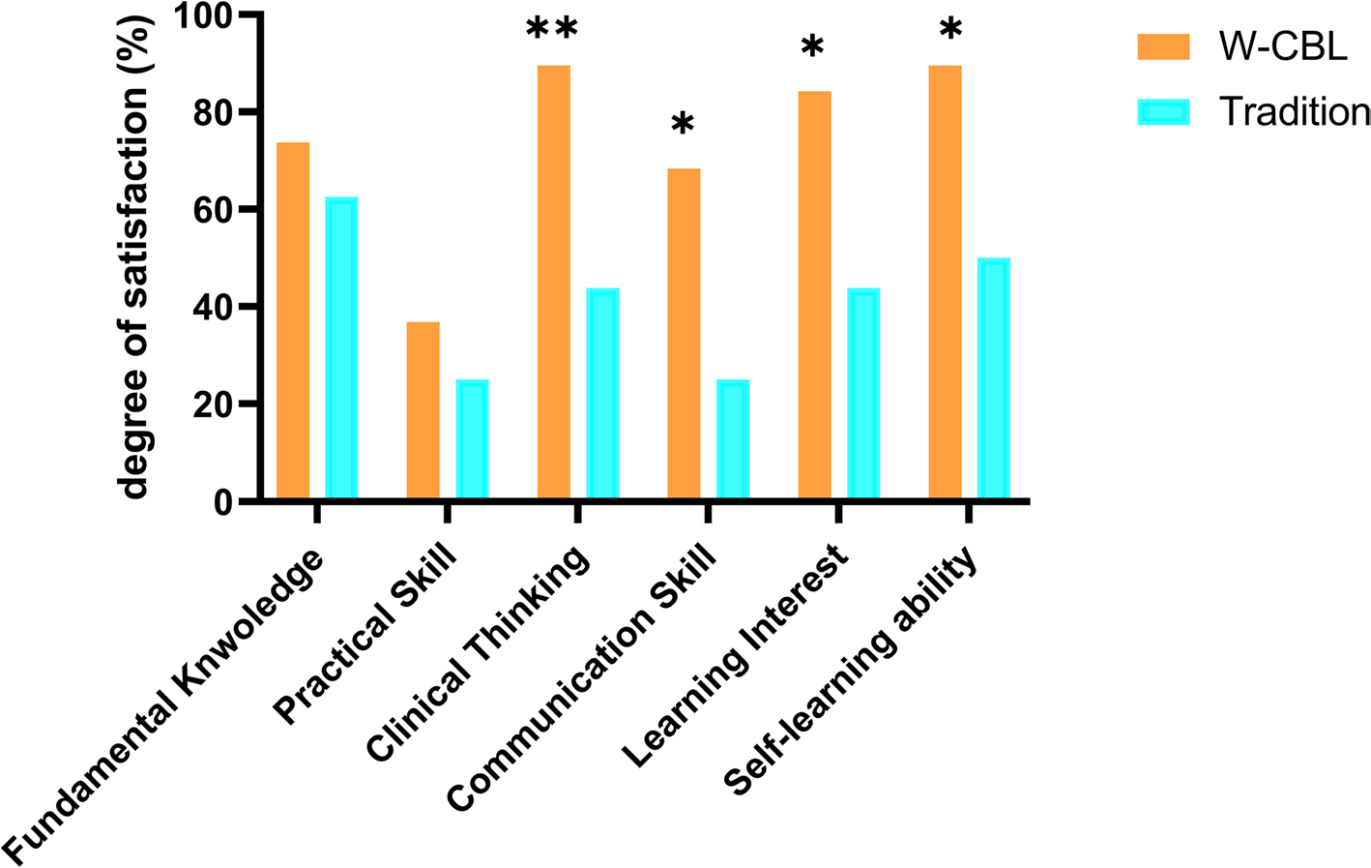
Comparison of the teaching satisfaction of the two groups of participants. The results of the questionnaire showed that residents in group W were more satisfied than group C in the aspects of “clinical thinking”, “communication skills”, “learning interest” and “self-learning ability” (89.5% vs. 43.8, 68.4% vs. 25.0, 84.2% vs. 43.8, 89.5% vs. 50%, all *P* < 0.05). However, there was no statistical difference in satisfaction between the two groups of residents in terms of fundamental knowledge and clinical practice skills

A total of 39 questionnaires (Survey A) distributed to all residents who have rotated in the Department of Anesthesiology from January 2018 to February 2021 at the end of the study and 34 questionnaires were recovered (W:C = 19:15), corresponding to a recovery rate of 87.2%. The results of the questionnaire showed that residents in group W were more satisfied in the aspects of “clinical thinking”, “communication skills”, “learning interest” and “self-learning ability”, and the difference was statistically significant (*P* < 0.05) (Fig. 1).

We conducted informal interviews with residents enrolled in this study to further understand the limitations of online CBL in clinical anesthesia teaching, and a total of 14 residents completed the interviews. Questions and answers of the interview are as follows.

Reasons residents do not prefer online CBL include: 1) Online CBL takes up too much time and energy: in addition to regular classes and focused discussions, new messages are always prompted in the WeChat group, and discussions may start at any time, which is not conducive to personal rest. 2) Information fragmentation: unlike lecture, the information in online CBL lacks focus and organization, requiring greater effort to screen and extract valid information. 3) Lack of realism to simulate clinical scenarios: Compared to bedside teaching, online lectures lack sufficient realism.

Factors that limit residents’ willingness to speak actively when participating in online CBL include: 1) Insufficient guidance by mentors. 2) Poor network conditions. 3) Unwillingness to follow voice messages resulted in some team members not being heard. 4) Unwilling to type. 5) Cannot check his/her phone very often due to trivial interruptions and therefore misses a lot of spontaneous discussions.

Residents believe that online CBL can help them in the following ways: 1) Enhanced the ability of information search. 2) Developed the habit of identifying, collecting and recording typical clinical cases. 3) Promoted the clinical reasoning and critical thinking ability. 4) Enhanced communication skills.

Some improvements to the online CBL are proposed, including: 1) Divide study time and rest time more precisely. 2) Develop WeChat mini programs for clinical simulation teaching to enhance the simulation of real-world clinical practice. 3) Automatically associate basic teaching materials such as up-to-date or text books.

## Discussion

CBL as a modern teaching mode is widely used in medical education [20, 21]. The learning goal of trainees is no longer the memorization of theoretical knowledge, but the practical application of mastering theoretical knowledge. However, in China, CBL has not been fully implemented and promoted due to the limitations of manpower, time and place. Educators have tried to optimize traditional CBL by improving teaching methods, such as Interprofessional education (IPE) and step-by-step (SBS) combined with CBL [20, 22]. Although much progress has been made in solving the shortage of teachers, there is still a lack of effective solutions to break through physical and temporal limitations of effective programs. In this study, we designed a new WeChat-based online CBL model in an attempt to compensate for the physical and temporal limitations of traditional CBL while maintaining its advantages.

### The advantages of W-CBL

First, WeChat provides a good platform for sharing educational resources and the latest information [23]. Group members may search for valuable information on the Internet and forward it directly to the group chat. At the same time, the treatment information of the case can be instantly uploaded to the group chat for other group members to review. The real-time sharing of information can effectively improve the efficiency and quality of discussions in CBL.

Second, traditional CBL requires participants to discuss in groups on a fixed schedule at a designated location, which is time-consuming [24, 25]. Online CBL, however, allows for asynchronous discussions and can take place “anytime, anywhere”, which improves the efficient use of participants’ time.

Third, traditional anesthesiology teaching tends to focus on applied teaching and lacks the integration of basic knowledge. When conducting online CBL teaching, the mentors required the students to explain the mechanism from the beginning when answering the guiding questions, and encouraged them to integrate the previous knowledge of anatomy, physiology, pathophysiology and pharmacology, so as to help them understand the “why” while learning the “how”. This largely makes anesthesiology more of a medical discipline than a technique. Residents also learned more about critical thinking of anesthesiology through online CBL.

Finally, group members’ resistance to speak during discussions is a major challenge in traditional CBL and may affect the final teaching effectiveness [26]. A study by Huang et al. [27] suggests that Microsoft can improve participant satisfaction and motivation in anesthesia teaching. Their data showed that in the new CBL model, team members were good listeners and communicators and were able to maintain a strong presentation motivation. Asynchronous communication in a virtual environment may be one of the reasons for the relaxed mindset of the trainees.

### The implementation conditions of online CBL

The core implementation conditions of online CBL include teaching facilities and teaching cases. In terms of teaching facilities, most other forms of online teaching approaches require equipment support such as computers, live/recording equipment, and specific teaching software [28]. In contrast, WeChat-based online CBL devices require only smartphones and Internet access, both of which tend to be common in China, and most medical education institutions have free wireless networks. The software requirement is WeChat App, the most widely available free social networking software in China [29], which is user -friendly and has been mastered by people of all ages [29]. Therefore, the facility requirements for WeChat-based online CBL are extremely low and conducive to widespread dissemination.

When it comes to teaching cases, the online CBL cases in this study were selected directly from the residents’ clinical work. The authenticity of the learning material motivates the trainees to explore and solve the problems involved, and at the same time, the trainees are able to have their medical protocols validated in further practice, which truly combines theory and practice. Compared to traditional CBL, online CBL is more efficient and diverse in case preparation [19], which helps to maintain the enthusiasm and motivation of trainees.

### Disadvantages of online CBL

First, residents had more freedom in receiving online CBL. This contributes to the intrinsic drive of trainees, but is not helpful in avoiding laziness and procrastination. Online CBL places higher demands on trainees’ self-discipline and time management skills. High-quality teaching emphasizes balancing freedom and discipline, and real-time supervision of the teaching process is one of the key challenges of online teaching.

Second, online CBL has limitations in ensuring the effectiveness of discussions. Without the constraints of a traditional classroom settings, participants can discuss topics of their own interest and may inadvertently stray from the topic, which may affect the motivation and activity of other participants. To overcome this drawback, mentors should always be actively involved in online discussions, encouraging non-speakers to discuss, ensuring that the discussion is on topic and prompting them to think more deeply. Other studies have also confirmed the need for teaching style training for participants and mentors before entering online CBL. Third, anesthesiology management belongs to clinical medical education, in which practice and operation play a crucial role in teaching. Thus, online CBL alone cannot meet the needs of trainees’ practical skills and face-to-face communication with patients. Currently, there is no teaching model that can completely replace bedside teaching in the process of clinical teaching for residents.

### Limitations

Firstly, this study was a single-center study, with a small sample size, and only residents who received training in our hospital were included. Further research will increase the number of teaching hospitals and carry out the teaching research of anesthesiology residents, primarily based on the Clinical Medical College, to expand the sample size. Meanwhile, the W-CBL standard teaching process of anesthesiology will be summarized for the convenience of large-scale promotion in the later stage. Secondly, in order to ensure the effectiveness and representativeness of the evaluation, the results of the study only used the classical scale to evaluate the teaching effect, but lacked the evaluation of the participants’ long-term working ability, as well as the embodiment of anesthesiology teaching. Further research will combine with entrustable professional activities (EPAs), an emerging clinical teaching assessment method, to track and assess students’ ability to perform clinical practice remotely, while enhancing clinical assessment of professional competence in anesthesia. Finally, this study did not use a strict sampling process, and all eligible students were included in the study. On the premise of sufficient sample size in the later stage, a randomized controlled study will be carried out to clarify the teaching advantages of W-CBL.

## Conclusions

Collectively, this study suggests WeChat, as China’s most widely used instant messaging APP, provides anesthesiologic residents with a favorable educational resource sharing platform and the latest academic information for clinical teaching. Furthermore, WeChat provides more novel and diversified cases resources for CBL, promoting more fully and in-depth discussions among residents, and the teaching process is not restricted by time and region. CBL teaching based on WeChat helps improve students’ theoretical performance, critical thinking and clinical decision-making. These advantages of CBL are an effective way of clinical teaching during the pandemic and may provide new teaching strategies for other clinical disciplines and regions.

## Data Availability

The datasets used and/or analyzed during the current study are available from the corresponding author on reasonable request.

## Abbreviations

LBL: Lecture-based learning
CBL: Case-based learning
W-CBL: WeChat combined with CBL
IPE: Interprofessional education
SBS: Step-by-step

## References

[1] Yan Y, Cheng X, Zhou C, Yang X, Li YQ (2021). The perceptions of anatomy teachers for different majors during the COVID-19 pandemic: a national Chinese survey. Med Educ Online.

[2] Hope C, Reilly JJ, Griffiths G, Lund J, Humes D (2021). The impact of COVID-19 on surgical training: a systematic review. Tech Coloproctol.

[3] Bi M, Zhao Z, Yang J, Wang Y (2019). Comparison of case-based learning and traditional method in teaching postgraduate students of medical oncology. Med Teach.

[4] Ilkiw JE, Nelson RW, Watson JL, Conley AJ, Raybould HE, Chigerwe M, Boudreaux K (2017). Curricular revision and reform: the process, what was important, and lessons learned. J Vet Med Educ.

[5] Dickinson BL, Lackey W, Sheakley M, Miller L, Jevert S, Shattuck B (2018). Involving a real patient in the design and implementation of case-based learning to engage learners. Adv Physiol Educ.

[6] Weidenbusch M, Lenzer B, Sailer M, Strobel C, Kunisch R, Kiesewetter J, Fischer MR, Zottmann JM (2019). Can clinical case discussions foster clinical reasoning skills in undergraduate medical education? A randomised controlled trial. BMJ Open.

[7] Gartmeier M, Pfurtscheller T, Hapfelmeier A, Grünewald M, Häusler J, Seidel T, Berberat PO (2019). Teacher questions and student responses in case-based learning: outcomes of a video study in medical education. BMC Med Educ.

[8] Khin-Htun S, Kushairi A (2019). Twelve tips for developing clinical reasoning skills in the pre-clinical and clinical stages of medical school. Med Teach.

[9] Chandrasekar H, Gesundheit N, Nevins AB, Pompei P, Bruce J, Merrell SB (2018). Promoting student case creation to enhance instruction of clinical reasoning skills: a pilot feasibility study. Adv Med Educ Pract.

[10] Clesham K, Piggott RP, Sheehan E (2020). A prospective review of a novel electronic journal club format in an orthopedic residency unit. J Surg Educ.

[11] Wu Q, Huang Y, Liao Z, van Velthoven MH, Wang W, Zhang Y (2020). Effectiveness of WeChat for improving exclusive breastfeeding in Huzhu County China: randomized controlled trial. J Med Internet Res.

[12] Zhang X, Chen X, Kourkoumelis N, Gao R, Li G, Zhu C (2021). A social media-promoted educational community of joint replacement patients using the WeChat app: survey study. JMIR Mhealth Uhealth.

[13] Rees S, Farley H, Moloney C (2021). How registered nurses balance limited resources in order to maintain competence: a grounded theory study. BMC Nurs.

[14] Yao L, Li K, He J, Liu L (2021). Pathophysiology teaching reform during the COVID-19 pandemic. Adv Physiol Educ.

[15] Luo P, Pang W, Wang Y (2021). WeChat as a platform for problem-based learning among hematological postgraduates: feasibility and acceptability study. J Med Internet Res.

[16] Li Y, Tse M (2020). An online pain education program for working adults: pilot randomized controlled trial. J Med Internet Res.

[17] Muthukrishnan SP, Chandran DS, Afreen N (2019). Planning, implementation, and evaluation of multicomponent, case-based learning for first-year Indian medical undergraduates. Adv Physiol Educ.

[18] Peñuela-Epalza M, De la Hoz K (2019). Incorporation and evaluation of serial concept maps for vertical integration and clinical reasoning in case-based learning tutorials: perspectives of students beginning clinical medicine. Med Teach.

[19] Zhang W, Li ZR, Li Z (2019). WeChat as a platform for problem-based learning in a dental practical clerkship: feasibility study. J Med Internet Res.

[20] Smith CJ, Matthias T, Beam E (2020). A mixed-methods evaluation of medical residents’ attitudes towards interprofessional learning and stereotypes following sonography student-led point-of-care ultrasound training. J Gen Intern Med.

[21] Shaw CV, D'Souza AJ, Cunningham R, Sarfati D (2020). Revolutionized public health teaching to equip medical students for 21st century practice. Am J Prev Med.

[22] Wei F, Sun Q, Qin Z, Zhuang H, Jiang G, Wu X (2021). Application and practice of a step-by-step method combined with case-based learning in Chinese otoendoscopy education. BMC Med Educ.

[23] Qiu Y, Qin H, Ying M, Xu K, Ren J (2020). WeChat-based health education to improve health knowledge in three major infectious diseases among residents: a multicentre case-controlled protocol. BMJ Open.

[24] Pan Y, Chen X, Wei Q, Zhao J, Chen X (2020). Effects on applying micro-film case-based learning model in pediatrics education. BMC Med Educ.

[25] Zhao W, He L, Deng W, Zhu J, Su A, Zhang Y (2020). The effectiveness of the combined problem-based learning (PBL) and case-based learning (CBL) teaching method in the clinical practical teaching of thyroid disease. BMC Med Educ.

[26] Rhodes A, Wilson A, Rozell T (2020). Value of case-based learning within STEM courses: is it the method or is it the student. CBE Life Sci Educ.

[27] Huang L, An G, You S, Huang S, Li J (2020). Application of an education model using the WeChat public platform in the standardized training of anesthesiology residents. Ann Palliat Med.

[28] Jaap A, Dewar A, Duncan C, Fairhurst K, Hope D, Kluth D (2021). Effect of remote online exam delivery on student experience and performance in applied knowledge tests. BMC Med Educ.

[29] Zhang L, Wei G, Xu Z, Huang Q, Liu G (2020). The prevalence of smartphones and WeChat use among older adults with chronic disease in a Western China. Comput Inform Nurs.

